# Detection of SARS-CoV-2 from combined nasal/rectal swabs

**DOI:** 10.1017/ash.2022.321

**Published:** 2023-01-09

**Authors:** Adriana M. Airo, Kevin R. Barker, Matthew P. Muller, Linda R. Taggart, Karel Boissinot, Ramzi Fattouh, Bridget Tam, Larissa M. Matukas

**Affiliations:** 1 Department of Laboratory Medicine and Pathobiology, University of Toronto, Toronto, Ontario, Canada; 2 Trillium Health Partners, Mississauga, Ontario, Canada; 3 Division of Infectious Diseases, Department of Medicine, Unity Health Toronto, Toronto, Ontario, Canada; 4 Department of Medicine, University of Toronto, Toronto, Ontario, Canada; 5 Division of Microbiology, Department of Laboratory Medicine, Unity Health Toronto, Toronto, Ontario, Canada

## Introduction

With over 500 million infections reported to date,^1^ severe acute respiratory syndrome coronavirus 2 (SARS-CoV-2) overwhelmed healthcare systems and remains a significant infection control priority in healthcare settings.

Previous studies assessed the utility of alternative clinical specimens (such as nasal swabs,^2^ stool^3^ and anal swabs^4^) for diagnosis of SARS-CoV-2 in the setting of swab scarcity or diagnostic screening due to prolonged shedding of SARS-CoV-2 RNA in stool.^5–7^ The objective of this study was to determine the performance characteristics of combined nasal/rectal (N/R) swab specimens used to screen new admissions for methicillin-resistant *Staphylococcus aureus* (MRSA*)* for the detection of SARS-CoV-2. If effective, this approach would provide a low-impact SARS-CoV-2 screening method at facilities that test new admissions for MRSA.

## Methods

### Study design

This study was conducted at two acute care hospitals affiliated with Unity Health Toronto (UHT), Canada, and was reviewed by the research ethics board at UHT. Patients admitted between March-April 2020 and December 2020-February 2021, which coincided with the peak of the 1^st^ and 2^nd^ COVID-19 waves in Toronto, respectively, were included in this study. At UHT, a double kit ESwab™ 493C02 (Copan Diagnostics, Murrieta, USA) containing two flocked swabs with liquid amies medium are used to screen for organisms of infection control significance on admitted patients using a risk-factor-based approach. One swab is used to circle inside the nares and the second swab is used to sample the sides of the rectum and perianal area.

### Laboratory testing

N/R swabs were transported at room temperature and stored at 4°C for up to seven days prior to testing. The specimens were heat-inactivated at 65°C for 30 min. Of note, we had a 5% RT-PCR inhibition rate (likely due to particulate matter in the specimen) that was resolved by 10-fold dilution using sterile liquid amies solution. The diluted specimen was centrifuged at 5,000 x g for 5 min and supernatant was transferred to a new tube. Nucleic acid extraction was performed with BD MAX™ ExK™ TNA-2 (Becton, Dickinson and Company, New Jersey, USA) and RT-PCR was performed using RealStar® SARS-CoV-2 RT-PCR Kit (Altona Diagnostics, Hamburg, Germany) according to the manufacturer’s instructions using the BD MAX™ System.

### Statistical Analysis

Statistical analyses were performed using GraphPad Prism software version 8.0 (GraphPad Software, San Diego, USA). To compare patient characteristics, disease severity, and mean age between patients with SARS-CoV-2 positive N/R swabs to those with negative N/R swabs, a Fisher’s exact test, chi-square test and an unpaired t-test were used, respectively. A *p*-value less than or equal to 0.05 (*p* ≤ 0.05) was considered statistically significant.

## Results

### Detection of SARS-CoV-2 in N/R swabs

The stability of SARS-CoV-2 RNA in N/R swabs was assessed. The cycle threshold (Ct) values from nine N/R swabs with detectable SARS-CoV-2 RNA were analyzed on day 0 and after storage for seven days prior to testing. The Ct-values of the SARS-CoV-2 targets (envelope and spike) were consistent at days 0 and 7 (data not shown).

Combined N/R swabs obtained from 100 inpatients who tested positive for SARS-CoV-2 by nasopharyngeal swabs (NPS) were included in the primary analysis. SARS-CoV-2 was detected in 39% of N/R swabs (Table 1). An additional 80 N/R swabs collected for screening of MRSA from inpatients with no clinical suspicion of COVID-19 were assessed to determine whether we could employ this as a routine surveillance strategy during COVID-19 surges. We did not detect SARS-CoV-2 in any of these specimens (data not shown). When compared with NPS, the sensitivity and specificity of N/R swabs was 39% and 100%, respectively. The positive and negative predictive value were 100% and 57%, respectively.

**Table 1.** Detection rate of SARS-CoV-2 RNA in N/R swabs collected from COVID-19 study inpatients. NPS and N/R specimens were processed at the Clinical Microbiology laboratory of UHT. Retrospective chart reviews were completed on seventy-five confirmed COVID-19 inpatients. Data was extracted from electronic medical record system using a standardized electronic abstraction form with defined extraction procedures. Demographics, clinical symptoms, and severity of disease in patients are shown, with the median age and interquartile range (IQR) or number of patients/swabs and percent (%). Data were obtained from the electronic medical record systems. Results from statistical tests (unpaired t-test^ψ^, Fisher’s exact test^†^ or chi-squared test^‡^) are shown. N/R: combined nasal/rectal.

	# of COVID-19 patients	SARS-CoV-2 RNA (+) N/R swab	SARS-CoV-2 RNA (-) N/R swab	*p*-value
Total	100	39 (39.0%)	61 (61.0%)	
Chart Review	75	28 (37.3%)	47 (62.7%)	
Age, y	63.9 (50.5-78.5)	63.5 (49.0-83.3)	65.0 (51.5-77.0)	>0.99^ψ^
Sex				
Men	51 (68.0%)	19 (37.3%)	32 (62.7%)	>0.99^†^
Women	24 (32.0%)	9 (37.5%)	15 (62.5%)	
Symptoms				
Fever	52 (69.3%)	21 (40.4%)	31 (59.6%)	0.30^†^
Dyspnea	46 (61.3%)	17 (37.0%)	29 (63.0%)	>0.99^†^
Cough	44 (58.7%)	20 (45.5%)	24 (54.5%)	0.13^†^
Nausea	13 (17.3%)	4 (30.8%)	9 (69.2%)	0.76^†^
Diarrhea	11 (14.7%)	2 (18.2%)	9 (81.8%)	0.31^†^
Vomiting	10 (13.3%)	3 (30.0%)	7 (70.0%)	>0.99^†^
Disease				
Asymptomatic	8 (10.7%)	2 (25.0%)	6 (75.0%)	0.15‡
Mild	23 (30.7%)	12 (52.2%)	11 (47.8%)	
Moderate	23 (30.7%)	5 (21.7%)	18 (78.3%)	
Severe or Critical	21 (28.0%)	9 (42.9%)	12 (57.1%)	

### Demographics of patients, symptoms, and severity of illness

Retrospective chart reviews were conducted on laboratory confirmed COVID-19 study inpatients (Table 1). No association was observed between the detection of SARS-CoV-2 in N/R swabs and reported gastrointestinal symptoms. To assess whether the detection of SARS-CoV-2 in N/R swabs was associated with disease severity in the same COVID-19 study cohort, patients were categorized as per disease severity according to WHO guidance.^8^ No association was observed between the patient’s severity of illness and the detection of SARS-CoV-2 in the N/R swab.

## Discussion

To our knowledge, this is the first study to assess the utility of combined N/R swabs for the detection of SARS-CoV-2. We focused our analysis on inpatients with laboratory confirmed COVID-19 and detected SARS-CoV-2 in 39% of N/R swabs.

Our data highlights several important findings. First, combined N/R swabs can be used to detect SARS-CoV-2; however, N/R swabs performed poorly compared with paired NPS. Second, our retrospective chart review analysis did not demonstrate an association between a patient’s disease severity and the detection of SARS-CoV-2 in the N/R swab, suggesting that a targeted approach for surveillance to a particular cohort may not be fruitful.

A major limitation of this study is the inability to evaluate if SARS-CoV-2 was shed from nasal or rectal collections; however, studies have reported high detection rates (> 80%) of SARS-CoV-2 from nasal swabs.^2^ In contrast, the detection rate of SARS-CoV-2 from faecal specimens is lower (25-67%).^3,4,6^ This discrepancy may be due to disparities in sampling time relative to symptom onset, specimen collection guidelines, and/or various chemistries used in nucleic acid extraction and RT-PCR. We did not assess the impact of sampling time relative to symptom onset on detection of SARS-CoV-2 in N/R swabs.

In conclusion, N/R swabs lack sensitivity and are insufficient as the sole specimen for the diagnosis of SARS-CoV-2.
